# A Copolymer With Lamellar Morphology^1^

**DOI:** 10.6028/jres.068A.023

**Published:** 1964-06-01

**Authors:** R. K. Eby

## Abstract

Electron microscopy, together with wide- and small-angle x-ray diffraction studies, indicates that copolymers of tetrafluorocthylene and hexafluoropropylene are lamellar. The lamellar development is extensive; the lamellae can be broad and extend for many microns. The perfluoromethyl groups are incorporated within the lamellae as point defects.

## 1. Introduction

Recent work on the structure of polymer crystals has suggested the hypothesis that crystalline polymers consist entirely of lamellae into which the molecules are systematically folded [1].^3^ In the extreme, this hypothesis implies that each molecule is completely within a crystal with no extensive interaction with any large amorphous regions. This in turn raises the possibility that some random copolymers may also be lamellar, with the comonomer units included in the crystal lattice as point defects. Indeed, there has been evidence for some time that random methyl groups do enter the polyethylene lattice [2, 3]. However, this evidence has not been universally admitted and it has been argued that exclusion of comonomer units from the crystal lattice would necessarily inhibit the development of lamellae in a copolymer [4].

The purpose of this paper is to present evidence that copolymers of tetrafluoroethylene and hexafluoropropylene are lamellar with the perfluoromethyl groups within the lattice as point defects. The same concepts were used by the author in earlier papers to present an analysis of the variation of copolymer transition temperature with comonomer ratio [5] and to describe the internal friction in copolymers of tetrafluoroethylene and hexafluoropropylene [6].

## 2. Experimental

Small-angle x-ray diffraction measurements were made on samples of copolymers with about 4, 11, and 17 mole percent hexafluoropropylene. Copper *Kα* radiation, slit collimation, a Rigaku-Denki diffractometer, and a proportional counter were used for most of the measurements. The diffraction curves were corrected for air scatter and backgrounds were established graphically. In a few cases a Kratky diffractometer was used with a Geiger counter; the effects of white radiation were eliminated by the use of balanced Co and Ni filters.

Wide-angle x-ray diffraction measurements of the same copolymers were made with a powder camera using chromium *Kα* radiation. The samples were cut with a square cross section of about 0.09 mm.^2^ The camera was flushed with helium; temperature was measured with a thermocouple at the helium inlet. The films on which the diffraction patterns were recorded were corrected for shrinkage.

Electron microscopy was used to examine exterior surfaces of the polymers. These surfaces were formed by crystallizing the same copolymers in contact with air. Chromium shadow replicas were prepared and were examined with a Phillips Model 100 electron microscope.

For optical microscopy, thin films were prepared by melting and spreading a small amount of polymer on a microscope slide placed on a hot plate. After the crystallization induced by removing the slide from the hot plate, these films were examined by reflected light and between crossed polaroids by transmitted light.

## 3. Results and Discussion

**Figure 1:**
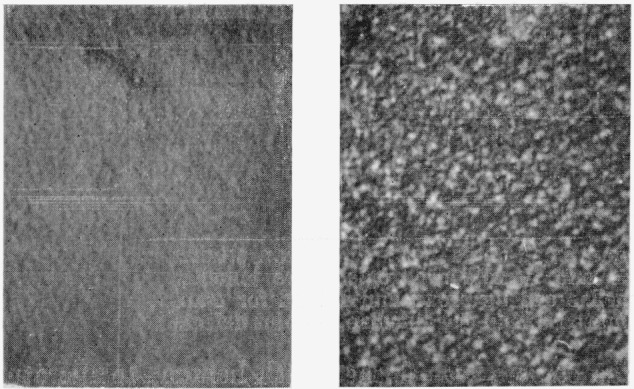
Optical micrographs of a thin film of the intermediate copolymer crystallized from the melt Left: reflected light; right: transmitted light with crossed polaroids.

**Figure 2:**
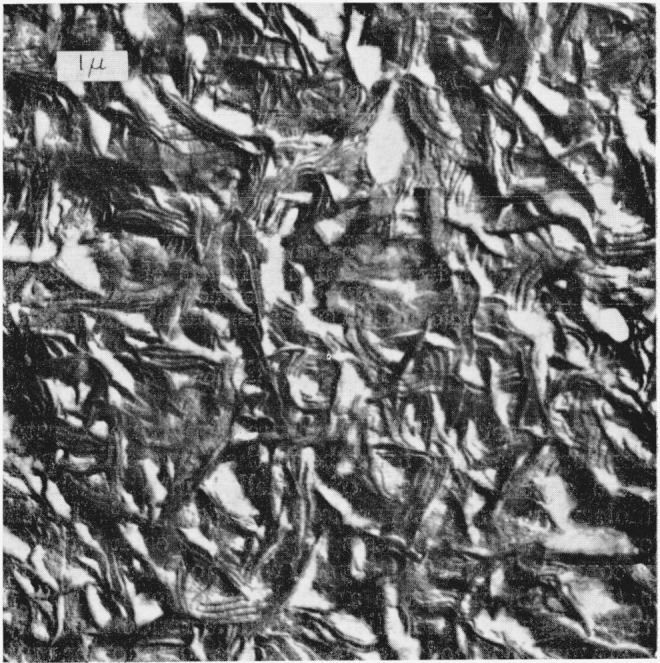
Electron micrograph of a surface similar to that in figure 1 Apparently lamellar structures can be seen.

**Figure 3:**
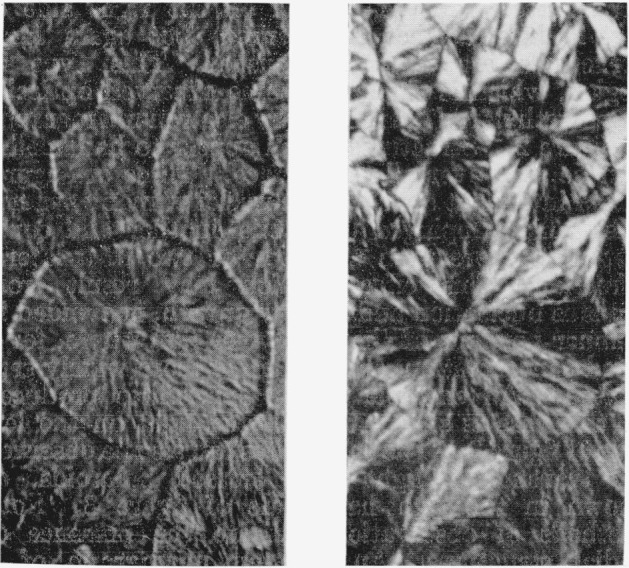
Optical micrographs showing spherulites in a thinner portion of the film in figure 1 Left: reflected light; right: transmitted light with crossed polaroids.

**Figure 4:**
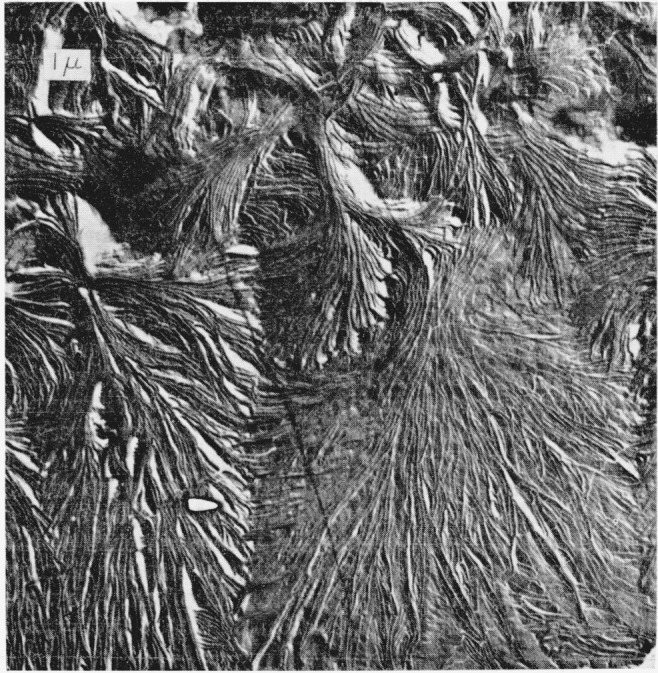
Electron micrograph of a surface similar to that in figure 3.

**Figure 5:**
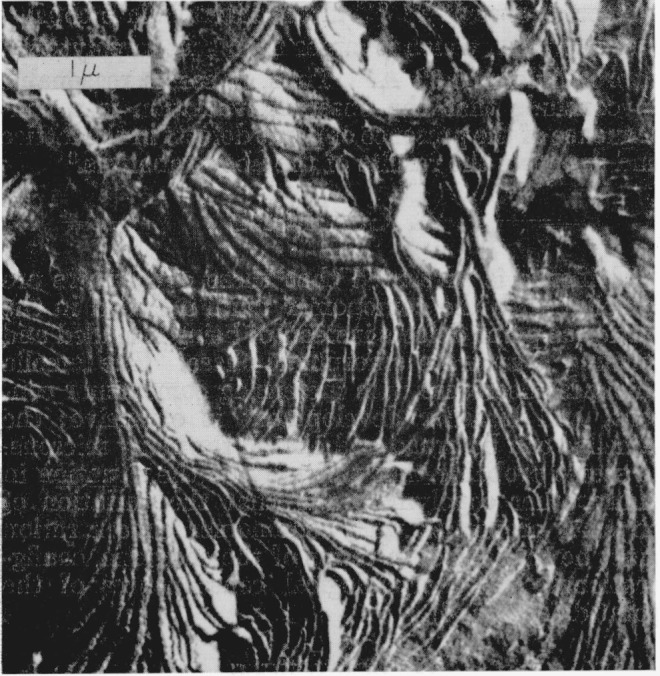
Enlargement of the upper portion of the electron micrograph in figure 4 Lamellar development can be seen. This figure also shows other structural features which are sometimes observed (right center).

**Figure 6:**
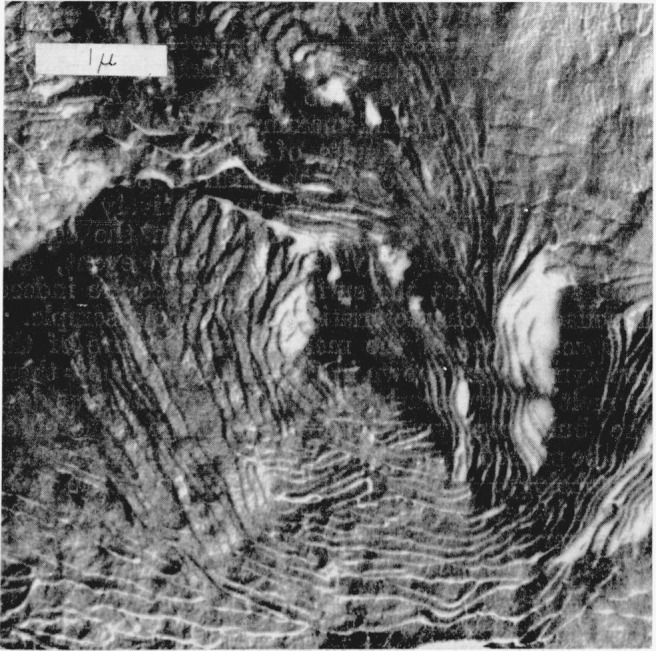
Electron micrograph of another region in which lamellar structures are apparent.

Optical micrographs of a typical thin-film preparation of the intermediate copolymer are shown in figure 1. On the left, the exterior surface is shown in reflected light. Small morphological features can be seen but there is no evidence of larger structures such as spherulites. Similarly, when this region is viewed between crossed polaroids by transmitted light (as shown on the right side of fig. 1) birefringence is observed, but again large structures are not distinguished. This type of region, which is similar to some observed optically in polyethylene [7], is shown by electron micrography in figure 2. Structures which appear to be lamellae can be seen over all the surface. These are aggregated into what appear to be nuclei of many spherulites which impinged upon one another and could not grow more than a micron or two in a lateral direction. This effect, which may result from copious nucleation and/or slow growth, is avoided in thinner portions of the copolymer film where, for a fixed concentration, the nuclei are more widely separated.^4^ An example of the spherulites produced in this case is shown by reflected light on the left side of figure 3. The spherulites can also be observed by transmitted light with crossed polaroids, as shown in the right-hand side of figure 3. Both methods of observation indicate the presence of radial structures. An electron micrograph of a similar surface is shown in figure 4. Here are seen the more fully developed spherulites characteristic of the thinner film portions. The radial lamellar development is extensive. Individual lamellae are broad and can be followed for many microns. The lamellar nature of the copolmyer is even more evident at higher magnifications as in figure 5 (an enlargement of the upper portion of fig. 4) and in figure 6. In the copolymers with fewer and more perfluoromethyl groups, the structure is similar except that the lamellae are thicker in the former and thinner and much less regularly developed in the latter.

Three questions may be asked about these apparently lamellar structures. Are they artifacts of the surface or are they lamellae and characteristic of the whole material? Are they anomalies associated with very low molecular weight components which might have been in the original polymer or might have been formed as degradation products? Are they composed of uninterrupted homopolymer sequences or are they truly copolymeric in composition?

**Figure 7:**
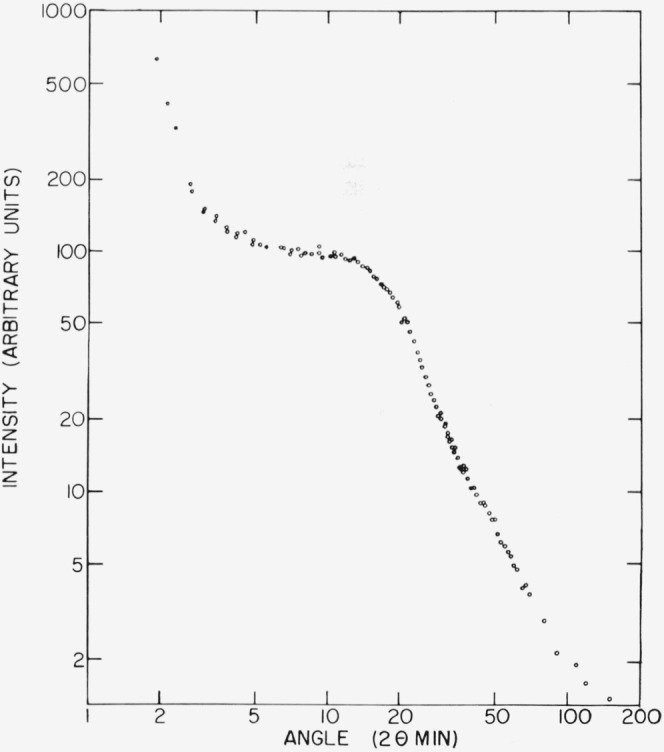
Intensity of diffracted x rays as a function of angle for copper Kα radiation and a piece of the intermediate copolymer cut from a bulk sample Only one diffraction maximum can be definitely identified.

Examination of the diffraction of x rays at small angles showed that samples similar to those used to obtain the micrographs in figure 1 produced diffraction maxima.^5^ The Bragg spacings calculated from these diffraction maxima are shown in table 1 where they are compared with the average dimensions of the lamellar structures measured on the electron micrographs. These agree to a degree which is better than should be expected considering the samples and techniques involved. More importantly, however, the data in table 1 show that as the hexafluoropropylene content is increased, the dimensions determined by electron microscopy and by x-ray diffraction decrease in the same fashion.^6^ Finally, a piece cut from the center of a bulk sample of copolymer exhibits a diffraction pattern similar to those obtained with the thinner samples. As figure 7 shows, this pattern, which was obtained with a Kratky diffractometer, exhibits only one maximum over a wide angular range. This result is different from that obtained with polyethylene [8] and indicates the absence of a second periodic structure with a period near the lamella thickness. While a few structures in figure 5 resemble the second structures observed in polyethylene [9], they appear to be larger and less regular than those in polyethylene and may be a consequence of material depletion at the surface. (Near 55 minutes—95Å–, there may be a small second maximum which would not correspond to the results of electron microscopy. This maximum, which is too small to be definitely identified in figure 7, would be relatively smaller and at a relatively larger angle than the second maximum of polyethylene.) In any event, the results show that the surface structures are indeed lamellae and characteristic of the whole sample.

Observations of the melting temperature of the copolymer films shown in figures 1 and 3 show them to melt at very nearly the same temperature as does the bulk copolymer. Also, the large spherulites can be melted and recrystallized in the more common morphology. These results indicate that the illustrated lamellar regions are not anomalies associated with very low molecular weight components segregated upon preparation of the films.

A statistical analysis based on a consideration of lamellar thickness and the concentration of random perfluoromethyl groups in each of these copolymers indicates that less than one percent of the material can be in homopolymer sequences as long as the lamellar thickness. (Orientation of the molecular axis with respect to the lamellae has not been uniquely determined. The birefringence of the spherulites is consistent with a predominantly tangential orientation of the axis. This observation is consistent with the axis being alined in the direction of lamella thickness. In any event, this alinement permits the largest statistical fraction of sufficiently long homopolymer sequences since the smallest lamella dimension is the thickness.) Even in the unlikely event that *all* these segments could be incorporated into lamellar crystals, they would contribute very little to small-angle diffraction and would not constitute enough material to account for the surface lamellae in two of the cases. In connection with this analysis, it should be noted that the difficulty of polymerizing polyhexafluoropropylene indicates that consecutive hexafluoropropylene units are not likely to occur in the copolymer [10]. Thus it seems that the lamellae contain the perfluoromethyl groups. As shown by figure 6 of reference 6, Stuart-Briegleb molecular models suggest that perfluoromethyl groups can enter the lattice and that the groups would resemble interstitial defects found in atomic solids. This would result in localized disturbance of adjacent molecular rows (shown schematically in figure 1 of reference 5) and should cause an increase in the associated lattice dimension [3]. Wide-angle x-ray diffraction measurements offer evidence for this. The separation of the molecular axes at 23 °C in the intermediate copolymer is 5.76 Å, which is larger than that in polytetrafluoroethylene, 5.66 Å.^7^ Thus, it seems reasonable to conclude that the observed variation is a consequence of the presence of point defects in the lattice and supports the results of the above analysis.^8^

## 4. Conclusion

Evidence is presented that these copolymers are lamellar with the comonomer units included in the lattice as point defects instead of being excluded and forming a separate amorphous phase. The lamella thickness decreases with increasing comonomer content, and lamellar development is extensive; the lamellae can be broad and extend for many microns. It seems probable that this may be the situation in many other copolymers and that the variation of physical properties with comonomer concentration must be thought of in terms of the energetic, configurational, and morphological consequences of the point defects [5, 6].

## Figures and Tables

**Table 1:** Comparison of lamella thicknesses determined by electron microscopy and x-ray diffraction

Electron micrographs	X-ray diffraction

*Å*	*Å*
420	450
335	285
260	230

## Footnotes

3 Figures in brackets indicate the literature references at the end of this paper.

4 Also, heating the molten copolymers to higher temperatures was found to reduce the concentration of nuclei.

5 These samples were slightly thicker and had a thermal history similar to that of the samples used in microscopy.

6 This decrease is similar to that which is observed with increasing methyl concentration in polyethylene [8]. Both these samples and the present ones were crystallized by cooling at some rate rather than isothermally.

7 It is difficult to make a correction for film shrinkage because the copolymer did not produce many lines at large angles. However, more accurate measurements using a diffractometer and an internal standard also show the separation of the molecular axes in the copolymer to be larger (R. K. Eby and L. H. Bolz, to be published). These measurements also show that the dimension of the copolymer is larger below the first order transitions. This eliminates the possibility that the copolymer dimension is larger at 23 °C merely because it is at a higher temperature with respect to its transition temperature than is polytetrafluoroethylene.

8 For even thinner lamellae (190 to 440 Å) the unit cell of polyethylene does not exhibit any detectable variation with lamella thickness [11]. This suggests that in this range lamella thickness does not influence unit cell dimension.
